# Broken-crescent sign at CT indicates impending aortic rupture in patients with acute aortic intramural hematoma

**DOI:** 10.1186/s13244-020-00880-9

**Published:** 2020-05-24

**Authors:** Sheung-Fat Ko, Chia-Yin Lu, Jiunn-Jye Sheu, Hon-Kan Yip, Chung-Cheng Huang, Shu-Hang Ng

**Affiliations:** 1 Department of Radiology, Kaohsiung Chang Gung Memorial Hospital and Chang Gung University College of Medicine, 123 Ta-Pei Road, Niao-Sung District, Kaohsiung, 833 Taiwan; 2 Department of Thoracic and Cardiovascular Surgery, Kaohsiung Chang Gung Memorial Hospital and Chang Gung University College of Medicine, Kaohsiung, Taiwan; 3 Department of Cardiology, Kaohsiung Chang Gung Memorial Hospital and Chang Gung University College of Medicine, Kaohsiung, Taiwan

**Keywords:** Acute aortic syndrome, Intramural hematoma, Aortic rupture, Computed tomography, Broken-crescent sign

## Abstract

**Background:**

This retrospective study evaluated the computed tomography (CT) features and clinical implications of a novel broken-crescent sign in patients with acute aortic intramural hematoma (IMH).

**Methods:**

Out of 104 patients with aortic IMH encountered in our institution between 2003 and 2018, nine patients exhibited a positive broken-crescent sign, which was defined as a focal defect within the hyper-attenuating crescentic IMH on unenhanced CT, corresponding to a smooth out-bulging of the aortic lumen on enhanced study. The clinical findings, CT features, and outcomes of these nine patients were analyzed.

**Results:**

Of five males and four females (age range 48–84 years, mean 69.7 years), six had type A and three had type B IMH. Five patients who had medical treatment and stable status for 1 to 3 days suffered sudden death, two of whom showed ascending aortic rupture with hemopericardium in one and adventitial tear with outward spillage of IMH in another at follow-up CT. The other four patients had early surgical or endovascular management survived; two demonstrated ascending aorta ecchymosis with adventitial tear and intact intima at surgery. Our results support the supposition that aortic IMH complicated with adventitial tear and partial outward seepage of IMH may generate a broken-crescent sign in CT. Despite initially stable clinical status, the residual intact inner aortic wall carries a high risk of sudden aortic rupture.

**Conclusions:**

In patients with acute aortic IMH, identification of a broken-crescent sign in CT is highly suggestive of impending aortic rupture, and early aggressive treatment is mandatory.

## Keypoints

- CT offers accurate diagnosis and classification of acute aortic intramural hematoma (IMH).
- IMH with adventitial tear and seepage of hematoma may generate a broken-crescent sign in CT.
- Positive broken-crescent CT sign is highly suggestive of impending aortic rupture.

## Background

Acute aortic syndrome (AAS), a potentially life-threatening aortic wall pathology, consists of acute aortic dissection (AAD) with an intimal flap and false lumen separating the aortic wall layers, intramural hematoma (IMH) with hemorrhage in the media without intimal defect, and penetrating atherosclerotic ulcer (PAU) with ulcer dissection into the media. The incidences of AAS range from 3.5 to 6.0 per 100,000 person-years, with AAD comprising 85–95% and IMH 5–10% of cases [1–3]. Classification and treatment methods for IMH are somewhat similar to AAD, with early surgery for type A and medical treatment for type B lesions [2–4]. However, IMH exhibits a more variable natural history. The progression of uncomplicated IMH appears to be more benign than that of AAD, and a wait-and-watch strategy with appropriate imaging follow-up may be a reasonable option [5–8]. Computed tomography (CT) is the modality of choice for the diagnosis and follow-up of AAS. CT features, including aorta dilation > 50 mm, maximum IMH thickness > 11 mm, and occurrence of ulcer-like projection (ULP) have been reported as predictors of aortic rupture in patients with IMH [3, 5, 9–11]. Moreover, we encountered two IMH cases (one type A and one type B) with stable clinical status under medical treatment who succumbed to sudden lethal cardiovascular collapse. Both cases demonstrated a smooth defect in the hyper-attenuating aortic IMH, with a broken crescent-like configuration and intact intima without contrast medium extravasation in the initial CT. This particular CT finding appears to be useful for predicting an aortic rupture in IMH patients. This retrospective study evaluated the CT features and clinical implications of a novel broken-crescent sign in patients with acute aortic IMH. A hypothesis on the development of this distinctive CT manifestation is postulated.

## Methods

From January 2003 to December 2018, among patients admitted to the emergency department of our institution due to acute chest discomfort with thoracic CT examinations, those with keywords included AAD, IMH, penetrating ulcer, or aortic aneurysm in the CT reports were collected from the picture archiving and communication system and their images were reviewed. Of them, patients who were suspected of having AAS were enrolled based on the following inclusion criteria: (1) presence of acute chest pain, back pain or intense chest discomfort, (2) definitive CT findings of IMH, and (c) except for those subjected to mortality, surviving patients were followed up for at least 12 months. IMH was diagnosed by the presence of regional crescentic or circular hyper-attenuating aortic wall thickening with longitudinal or circumferential extension along the aortic wall in a non-spiral fashion on unenhanced CT and the absence of intimal disruption, a dissecting flap, or enhanced false lumen on enhanced study. Patients with only unenhanced CT, poor CT image quality due to motion artifacts, refusal of any treatment against advice discharge, or loss of follow-up were excluded and eventually a total of 104 patients were enrolled.

Thoracic CT examinations were performed with a variety of facilities included 4-detector row scanners (Somatom Volume Zoom, Siemens; Lightspeed, GE Healthcare), 64-detector row scanners (Somatom Definition AS, Siemens; Acquilion 64, Toshiba Medical Systems), and dual-source scanner (Somatom Definition Flash, Siemens). Each examination included unenhanced and contrast material enhanced acquisitions with a scanning range from the thoracic inlet down to the kidney or lower pelvic level. Electrocardiographic gating was not used. Nonionic contrast medium (350 mgI/mL) was administered with a bolus injection (rate, 2–4 mL/s; volume, 70–100 mL) by using a power injector. Scanning was triggered with a bolus-tracking method with a threshold value of 120 HU in the aortic arch. Scanning parameters included field of view 300–350 mm, matrix 512 × 512, 100–130 kVp, 150–280 mAs, rotation time 0.5 s, spiral pitch factor 0.6–1.2, exposure modulation (+), and reconstruction at 2–5-mm-section thickness. The coronal and sagittal oblique view was also reconstructed for assessment.

All CT images of these 104 patients were independently reviewed by two experienced thoracic radiologists (28 and 18 years in thoracic imaging, respectively) blinded to the clinical outcome to search for the so-called broken-crescent sign. Broken-crescent sign was defined as the presence of a focal central or eccentric defect with obtuse edges in the hyper-attenuating crescentic or circular IMH on unenhanced CT and the defect corresponded to a smooth focal out-bulging of aortic lumen on subsequent enhanced study. Definitive differentiations of broken-crescent sign from PAU, ulcer-like projection (ULP), or intramural contrast pools were specifically performed.

Of 104 patients, IMH afflicted the ascending aorta and/or the aortic arch in 46 patients (type A, 44.2%), whereas IMH afflicted only the descending aorta beyond the orifice of the left subclavian artery in the remaining 58 patients (type B, 55.8%). The treatment modality and outcomes of these 104 patients were recorded with an averaged follow-up duration of 25.8 months. Among 104 patients, broken-crescent sign was identified in nine patients by both reviewers with no interobserver variability. The medical records of these nine patients were scrutinized for demographics, clinical manifestations, known past diseases, details of medical and surgical treatment, and final outcomes. Our institutional review board approved this study (approval number: 202000207B0). The need for written informed consent from the patients was waived by the board due to the retrospective and anonymous nature of the analysis.

## Results

### In-hospital mortality and 1-year mortality of aortic IMH

**Fig. 1:**
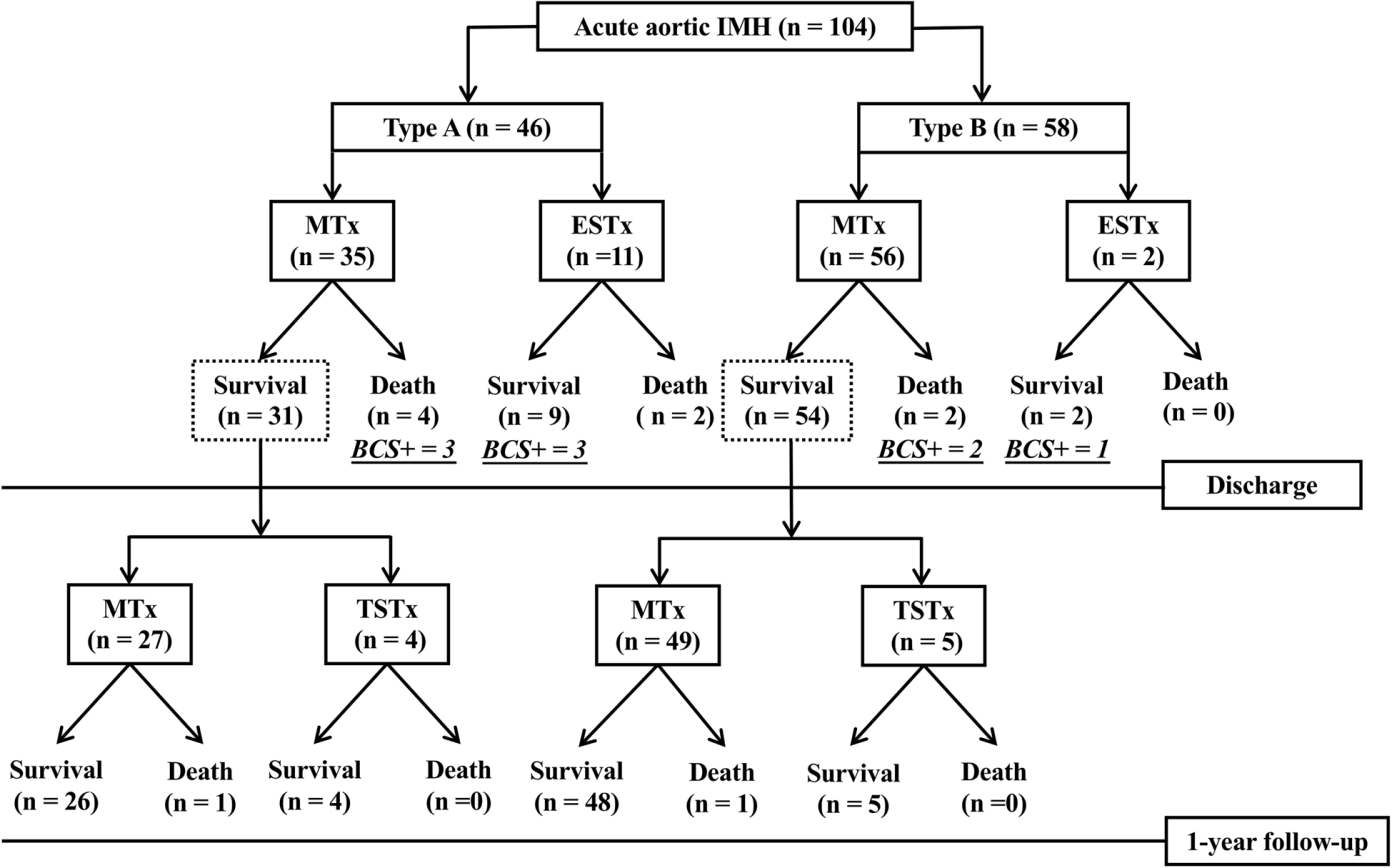
Selected treatment and outcomes of 118 patients with acute aortic IMH. MTx = medical treatment, ESTx = early surgical or endovascular treatment, BCS+ = positive broken-crescent sign on CT, TSTx = timely surgical or endovascular treatment

The treatment methods and clinical outcomes of 104 patients with acute aortic IMH are summarized in Fig. 1. Thirty-five of 46 patients (76.1%) with type A and 56 of 58 patients (96.6%) with type B, uncomplicated IMH received initial medical treatment; the in-hospital mortalities were 11.4% (4/35 patients) and 3.4% (2/58 patients), respectively. Thirteen patients with complicated IMH, managed with early surgical or endovascular treatment, had in-hospital mortalities of 18.2% (2/11 patients) in type A and 0% (0/2 patients) in type B IMH, respectively. Overall in-hospital mortality was 7.7% (8/104 patients).

Among 85 patients with medical treatment and uneventful discharges, four type A (12.9%, 4/31 patients) and five type B IMH (9.3%, 5/54 patients) showed progression to aortic dissection or dilatation, development of ULP, or persistent chest or back pain in follow-up and underwent subsequent surgical or endovascular treatment. Of these 85 patients, death occurred in one type A (3.7%, 1/27 patients) and one type B (2.0%, 1/49 patients) IMH patient with medical treatment. With the adoption of a wait-and-watch strategy for initially uncomplicated aortic IMH, 1-year mortalities after medical treatment and timely surgery were 14.3% (5/35 patients) in patients with type A and 5.4% (3/56 patients) in patients with type B IMH with an overall mortality of 9.6% (10/104 patients).

#### Clinical features of nine patients with broken-crescent sign

Among 104 patients with IMH, nine patients had positive broken-crescent CT sign and their clinical details are summarized in Table 1. There were five male and four female patients, ranging in age from 48 to 84 years (mean 69.7 years). The mean age of the mortality group seemed older than the survival group (mean 73 years vs 66 years). Eight patients had hypertension, but there was no substantial difference between the mortality and survival groups (mean 188/123 mmHg vs 182/117 mmHg). Initial electrocardiograms were unremarkable. Elevation of serum blood sugar levels in two patients, mild anemia in two patients, and mild leukocytosis in four patients were noted, but other laboratory data was noncontributory. The serum levels of cardiac troponin and creatine kinase-myocardial band isoenzyme were within normal ranges.

**Table 1:** Summary of 9 patients with aortic intramural hematoma (IMH) and broken-crescent sign on chest CT

Case no.	Age	Sex	Clinical findings	Co-morbidities	IMH type/thickness/max. aorta diameter	Time to surgery	Surgical findings and treatment	Follow-up
1	84	M	ACP and dizziness lessen after 5 hrs, stable for 2 days with sudden CV collapse, follow-up CT revealed aortic rupture	Gout, HT, hyperlipidemia	A/4 mm/52 mm	NA	NA	Death
2	80	M	Type B IMH stable for 2 days with recurrent chest pain and dyspnea; follow-up CT revealed blood clot abutting aorta and pleural effusion	HT, gastric cancer post gastrectomy	B/8 mm/47 mm	54 hrs	Shock just before endovascular repair	Death
3	62	M	ACP with radiation to back alleviated after 4 hrs, stable for 1 day with sudden CV collapse	HT	A/5 mm/48 mm	NA	NA	Death
4	55	M	ACP alleviated after 1 day, stable for 3 days with sudden CV collapse	HT	A/6 mm/46 mm	NA	NA	Death
5	83	F	ACP alleviated after 6 h, sudden CV collapse on day 2	HT	B/5 mm/42 mm	NA	NA	Death
6	48	F	Chest tightness and dyspnea for 3 hrs	DM, angina, HT	A/4 mm/48 mm	8 hrs	AsAo ecchymosis with adventitial tear, AsAo graft	4 yrs, stable
7	77	F	ACP radiation to back alleviated after 4 hrs	HT, left knee replacement	A/4 mm/46 mm	9 hrs	AsAo ecchymosis with adventitial tear, AsAo graft	3 yrs, stable
8	66	M	ACP for 2 hrs with cold sweating	DM, HT, CKD, arrhythmia	A/6 mm/53 mm	7 hrs	Impending rupture, AsAo graft	3 yrs, stable
9	72	F	Severe back pain for 2 hrs	CKD, dialysis	B/7 mm/43 mm	9 hrs	Endovascular repair	5 yrs, stable

*ACP* acute chest pain, *AsAo* ascending aorta, *CKD* chronic kidney disease, *CV* cardiovascular, *DM* diabetes mellitus, *F* female, *hrs* hours, *HT* hypertension, *M* male, *max*. maximum, *NA* not applicable, *yrs* years

**Fig. 2:**
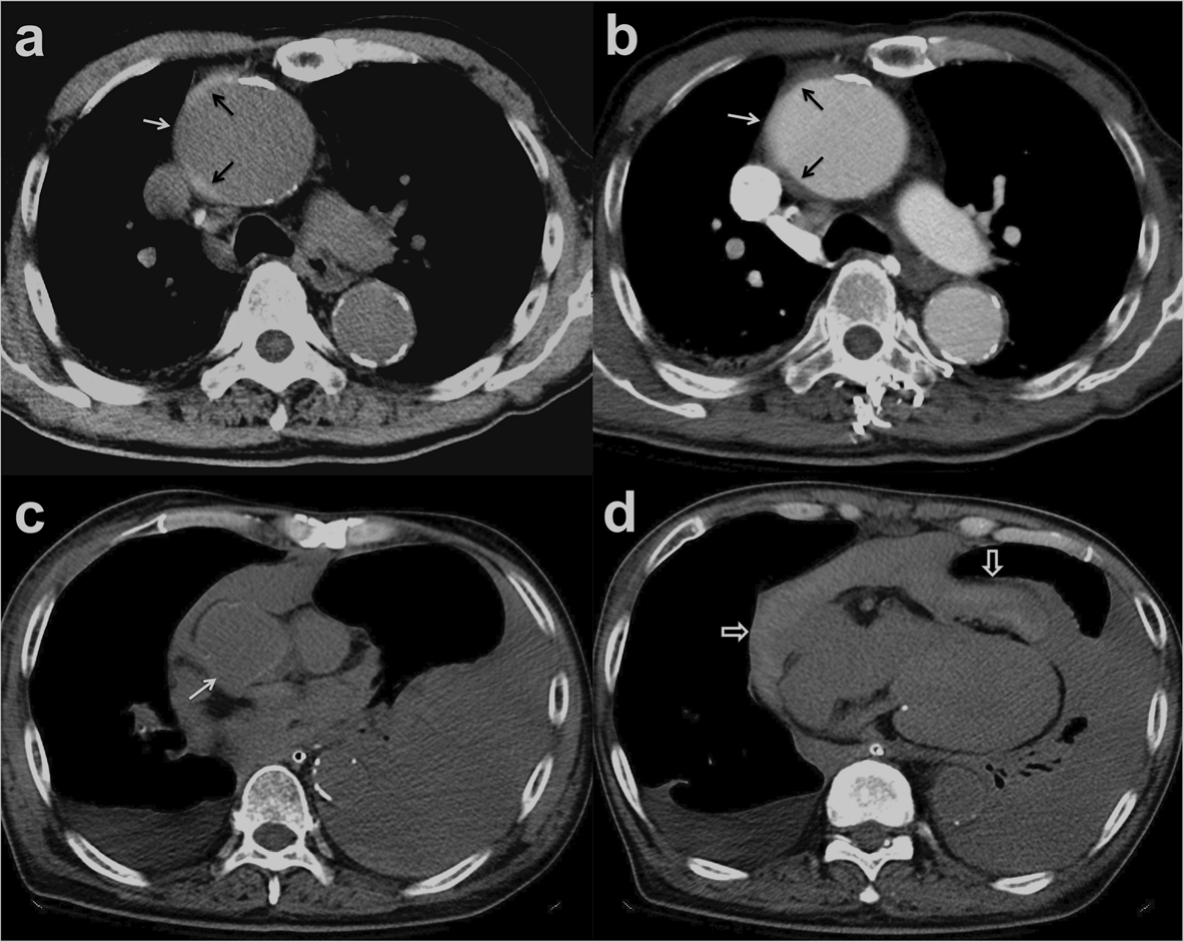
**a** Unenhanced axial CT shows a smooth central defect (white arrow) in the hyper-attenuating crescentic hematoma (black arrows) along the ascending thoracic aorta. **b** Enhanced CT shows a focal out-bulging (white arrow) of the aortic lumen, with smooth obtuse edges corresponding to the defect on unenhanced study and non-enhancing crescentic hematoma (black arrows). Note the absence of intimal flap or contrast medium extravasation. **c**, **d** Unenhanced follow-up CT shows rupture of the ascending aorta (white arrow), hemopericardium (open arrows), and left pleural effusion

**Fig. 3:**
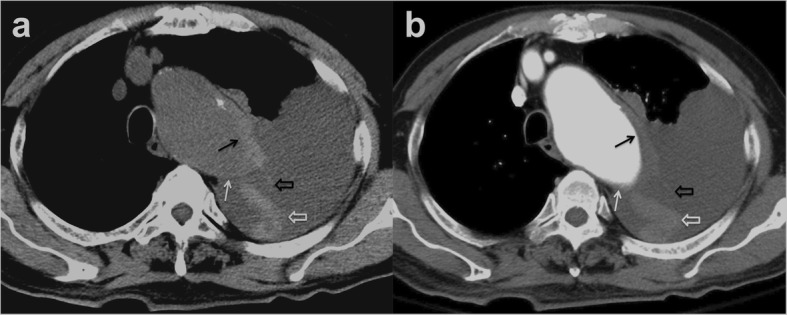
**a** Unenhanced axial CT shows a smooth eccentric defect (white arrow) in the hyper-attenuating crescentic hematoma (black arrow) along the descending thoracic aorta and (**b**) corresponding focal out-bulging (white arrow) of the aortic lumen with smooth obtuse edges in enhanced study, outward displacement of partially torn adventitia (open black arrow), dislodged blood clot (white open arrow), and left pleural effusion. Note the absence of intimal flap or contrast medium extravasation

**Fig. 4:**
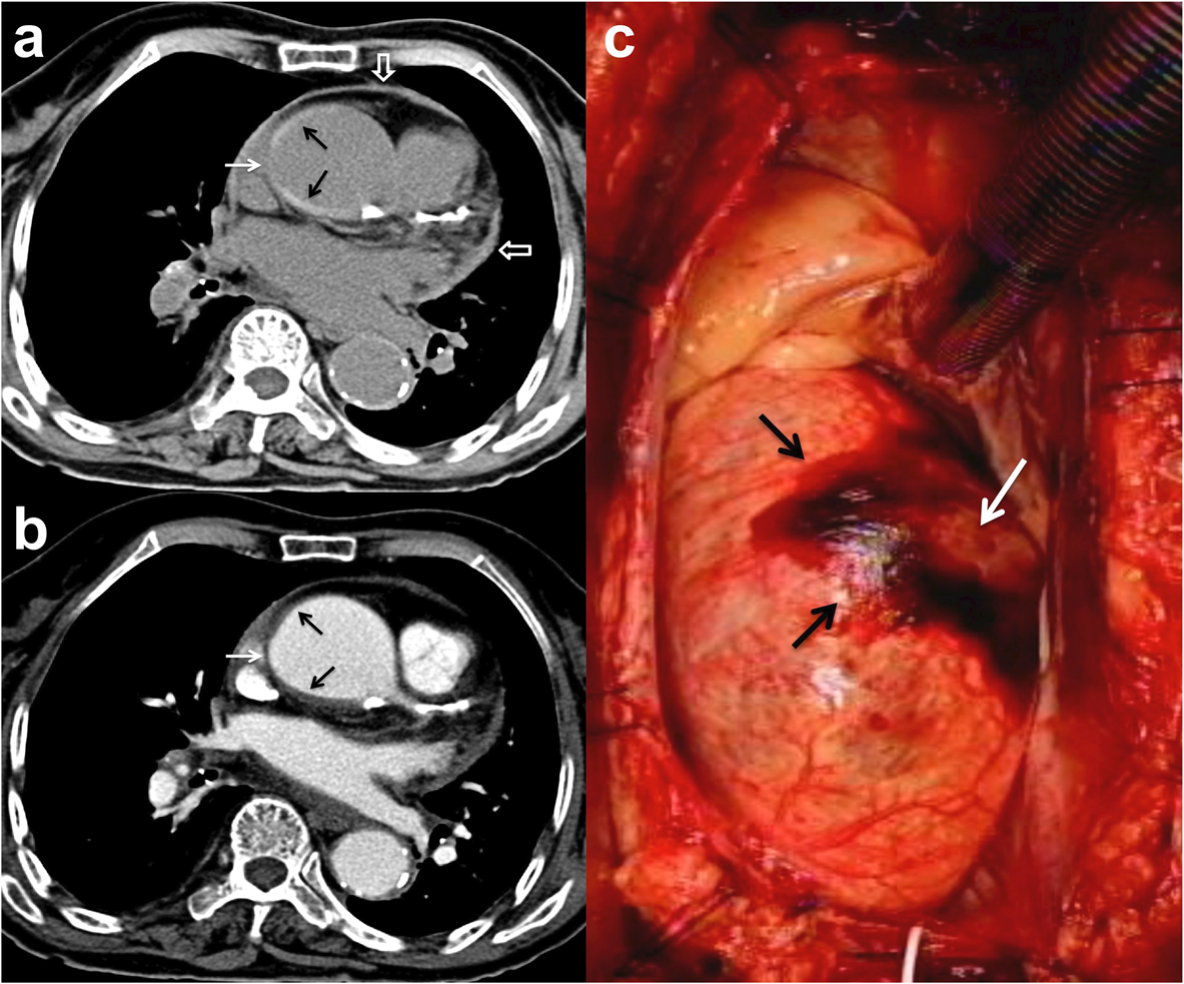
**a** Unenhanced axial CT shows a smooth central defect (white arrow) in the hyper-attenuating crescentic hematoma (black arrows) along the ascending thoracic aorta and minimal hemopericardium. **b** Enhanced CT shows a smooth focal out-bulging (white arrow) of the aortic lumen corresponding to the defect on unenhanced study and non-enhancing crescentic hematoma (black arrows). Note the absence of intimal flap or contrast medium extravasation. **c** Surgical findings confirmed ecchymosis at the ascending aorta caused by IMH and focal aortic adventitial defect

Patients 1 to 5 had initial medical treatment and stable status for 1 to 3 days suffered sudden death. Patient 1 with type A IMH (Fig. 2a, b) had sudden cardiovascular collapse 2 days later, and follow-up CT revealed aortic rupture and hemopericardium (Fig. 2c, d). Patient 2 with type B IMH had recurrent symptoms after 2 days, and follow-up CT showed adventitial tear with partial outward spillage of hematoma (Fig. 3). Patients 6 to 9 had early surgical or endovascular management recovered uneventfully. Patients 6 and 7 demonstrated ascending aorta ecchymosis with adventitial tear and intact intima at surgery (Fig. 4), and patient 8 had IMH with impending ascending aortic rupture. Pathologic examinations confirmed aortic IMH.

### Imaging features in patients with broken-crescent sign

A definitive CT diagnosis of aortic IMH was established based on the presence of hyper-attenuating crescentic aortic wall thickening on unenhanced CT. In a retrospective review, all nine patients exhibited a distinctive, positive broken-crescent sign in CT, with a smooth focal defect in IMH that corresponded to a smooth out-bulging of the aortic lumen with obtuse edges on the subsequent enhanced study (Figs. 2, 3, and 4). All nine patients demonstrated smooth aortic lumen with neither intima flap nor contrast medium extravasation, indicating an intact inner wall of the aorta. There were no substantial differences in maximum aortic diameter (range 42–52 mm, mean 47.0 mm vs. range 43–53 mm, mean 47.5 mm) or maximum IMH thickness (range 5–8 mm, mean 5.6 mm vs. range 4–7 mm, mean 5.3 mm) between the deceased (5/9) and surviving (4/9) patients, respectively. Of note, among eight out of 104 patients with in-hospital mortality, broken-crescent sign was retrospectively identified in five patients. Of these eight patients, three demonstrated minimal hemopericardium in CT but no other specific imaging sign for prediction of dismal outcome could be sought. Furthermore, there were no significant differences in maximum aorta diameter and maximum IMH thickness between in-hospital mortality and survival groups (49.4 ± 7.1 mm vs 46.8 ± 11.8 mm, *p* = 0.571; and 8.8 ± 4.2 mm vs 9.1 ± 5.8 mm, *p* = 0.373, respectively) (Mann-Whitney test).

## Discussion

Aortic IMH is generally considered to be caused by rupture of the vasa vasorum, with the hematoma appearing in the exterior part of the media near the adventitia without rupture into the aortic lumen. As seen in the present study, most patients with IMH presented with chest and back pain indistinguishable from AAD, with predisposing hypertension [1–4]. Although classifications and prognosis of aortic IMH are similar to those of AAD, the optimal management of acute IMH is less widely accepted than that of classic AAD. Such uncertainty is caused by the variable natural history of aortic IMH, ranging from spontaneous regression of the hematoma to development of ULP, aortic dissection, aneurysm, or even aortic rupture [5–8].

The management of IMH depends on the clinical status of the patient and Stanford classification. Emergency surgery is indicated in type A IMH complicated with pericardial effusion, peri-aortic hematoma, large aneurysms, or malperfusion with a high mortality rate (11–24%) [2–4, 12]. Most type B IMH can be managed medically with an in-hospital mortality risk of 7–10%; endovascular repair or surgery would be considered in complicated type B IMH with aortic dilatation, ulcer-like projection (ULP), and persistent or recurrent symptoms [2–4, 13]. Among our patients who underwent early surgery, the in-hospital mortalities were 18.2% and 0% in type A and type B IMH, respectively.

The optimal initial treatment for uncomplicated type A IMH still remains to be explicated. Several studies advocated immediate surgical therapy for type A IMH, owing to possible progression to aortic rupture or dissection, high mortality (40%) in medically treated patients, and high frequency (30–40%) of eventual surgery due to complications [2–4, 12–14]. Moreover, the outcomes of the medically treated patients with conversion to surgery caused by newly developed adverse events may be poor due to hemodynamic instability. Instead, early surgery for patients with uncomplicated type A IMH should be much easier with less-critical complications [13, 14]. In contrast, some studies suggested that medical treatment and timely surgical repair can be a rational strategy for uncomplicated type A IMH [6–8, 15]. IMH differs from AAD by virtue of the absence of blood flow within the hematoma and up to 67% of patients with medically treated type A IMH exhibited spontaneous regression. The risk of unexpected deterioration or sudden death versus the risk of emergency aortic surgery should be weighed carefully [7, 8, 15, 16]. Consistent with several prior studies by adopting such a wait-and-watch policy, our results showed favorable clinical outcomes, with an overall in-hospital and 1-year mortality of 7.7% and 9.6%, respectively. Of note, recent improvements in surgical techniques for AAS result in low mortality even in delayed surgery [6, 17]. In the present study, no mortality was noted in 9 patients who underwent timely surgery.

Magnetic resonance imaging (MRI) and CT are the gold standards for the diagnosis and classification of aortic IMH. Although MRI is advantageous in providing high contrast-resolution images and signal characterization of the age of the hematoma without radiation exposure; limited availability and long examination impede its role as a first-line modality for acutely sick patients with IMH [2, 3, 18]. Besides 96–100% sensitivity and 100% negative predictive value, widespread availability, high spatial resolution, and full anatomic assessment of the whole aorta within a short duration make CT the preferred tool for diagnosing IMH [3, 5, 17–19]. Unenhanced CT is crucial for delineating the hyper-attenuating crescentic aortic wall thickening with longitudinal or circumferential propagation in a non-spiral fashion. Enhanced CT is useful for confirmation of the absence of a dissection flap; identification of PAU and ULP, which may portend poor prognosis; monitoring of an intramural blood pool, which is usually self-limited; and measurement of the aortic diameter and IMH thickness [5, 16–18]. The maximum aortic diameter of ≥ 50 mm has been reported as the most powerful predictor of IMH complications [3, 9, 16]. A mean maximum aortic diameter of 47.3 mm was noted in the present study, and only two patients (22%) had an aortic diameter of ≥ 50 mm.

To our knowledge, the application of a broken-crescent sign as a CT predictor of impending aortic rupture in patients with acute aortic IMH has not been previously reported. Differentiation of the broken-crescent sign of IMH from PAU and ULP in CT is imperative. CT of a PAU typically appears as a contrast-enhanced outpouching with acute jagged edges penetrating into the media of the aortic wall, mostly in the thoracoabdominal aorta with severe atherosclerotic disease [5, 19]. ULP is a localized, contrast-enhanced pouch in the IMH with obvious communication with the true lumen, mostly found in the ascending aorta and the aortic arch at points of great mechanical stress [5, 11, 19]. The broken-crescent sign is characterized by a smooth focal defect with obtuse edges in the hyper-attenuating crescentic hematoma, corresponding to a focal out-bulging of enhanced aortic lumen with no contrast medium leakage.

**Fig. 5:**
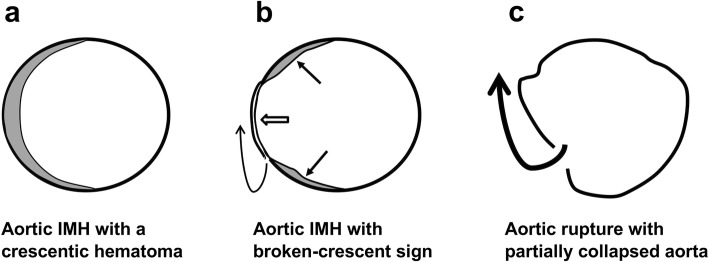
Sketch shows the postulation of the development of broken-crescent sign in CT. **a** Acute aortic IMH with typical hyper-attenuating crescentic hematoma along the aorta on axial CT. **b** Partial aortic adventitial tear with outward seepage of the hematoma (curve arrow), leading to a broken-crescent configuration (arrows). Focal out-bulging (open arrow) of the residual inner aortic wall results in temporary sealing of the underlying adventitial defect. **c** Rupture of the aorta through the weakened residual inner aortic wall (curve arrow) with partially collapsed aorta

The hyper-attenuating crescent sign is seen in unenhanced CT as a localized curvilinear hyper-attenuating zone within the thrombus of abdominal aortic aneurysms and is an early sign of acute or impending rupture. It is caused by intrathrombotic hemorrhage and dissection of blood into the peripheral thrombus or aortic wall with the weakening of the support structure of the aneurysm incurring vulnerability to rupture [19, 20]. On the other hand, broken-crescent sign in IMH seems to exhibit different pathophysiological changes of the aortic wall. We postulate that acute aortic IMH may complicate with a subtle adventitial tear followed by partial outward seepage of an intramural blood clot, leading to a broken-crescent configuration on CT (Fig. 5). The CT features in patient 2 and surgical findings in patients 6 and 7 supported this postulation. Because the residual inner aortic wall remained intact, there was no contrast medium extravasation. Due to local wall thinning, focal outward bulging of the aortic lumen may have compressed on and sealing up the underlying adventitial tear temporarily; thus, the IMH spillage could be transiently alleviated, resulting in a short duration of clinical mitigation. More importantly, with such a weakened residual inner aortic wall at the site of the broken crescent, IMH patients might subject to a high risk of subsequent aortic rupture, as seen in the follow-up CT of patient 1. Notably, in our patients with positive broken-crescent sign ascribed to outward seepage of an IMH, the maximum IHM thickness (range 4–8 mm, mean 5.4 mm) was far thinner than the threshold (11 mm) used for predicting IMH complications [9, 10].

Our study had some limitations. First, since an IMH with a positive broken-crescent sign is infrequent, our study had a small number of patients. Second, the study sample was heterogeneous and included various CT facilities ranging from conventional to 256-multidetector CT, because it took a long time to collect such uncommon cases. Third, this study was retrospective and we were not able to perform pathologic correlations with CT. Fourth, even though CT is considered the modality of choice for assessing AAS, the present study lacks other imaging for comparison.

## Conclusions

In patients with acute aortic IMH, identification of a broken-crescent sign in CT is highly suggestive of impending aortic rupture, and early surgical or endovascular treatment is mandatory.

## Data Availability

The datasets used and/or analyzed during the current study are available from the corresponding author on reasonable request.

## Abbreviations

AAD: Acute aortic dissection
AAS: Acute aortic syndrome
CT: Computed tomography
IMH: Intramural hematoma
PAU: Penetrating atherosclerotic ulcer
ULP: Ulcer-like projection
